# Loosely Coupled GNSS and UWB with INS Integration for Indoor/Outdoor Pedestrian Navigation †

**DOI:** 10.3390/s20216292

**Published:** 2020-11-05

**Authors:** Vincenzo Di Pietra, Paolo Dabove, Marco Piras

**Affiliations:** 1 Department of Environment Land and Infrastructure Engineering (DIATI), Politecnico di Torino, Corso Duca degli Abruzzi 24, 10129 Torino, Italy; paolo.dabove@polito.it (P.D.); marco.piras@polito.it (M.P.); 2 Politecnico di Torino Interdepartmental Centre for Service Robotics (PIC4SeR), Politecnico di Torino, Corso Duca degli Abruzzi 24, 10129 Torino, Italy

**Keywords:** UWB, GNSS, indoor positioning, INS, pedestrian navigation, sensor integration, data fusion

## Abstract

The growth of location-based services (LBS) has increased rapidly in last years, mainly due to the possibility to exploit low-cost sensors installed in portable devices, such as smartphones and tablets. This work aims to show a low-cost multi-sensor platform developed by the authors in which an ultra-wideband (UWB) indoor positioning system is added to a classical global navigation satellite systems–inertial navigation system (GNSS-INS) integration, in order to acquire different synchronized data for further data fusion analysis in order to exploit seamless positioning. The data fusion is based on an extended Kalman filter (EKF) and on a geo-fencing approach which allows the navigation solution to be provided continuously. In particular, the proposed algorithm aims to solve a navigation task of a pedestrian user moving from an outdoor space to an indoor environment. The methodology and the system setup is presented with more details in the paper. The data acquired and the real-time positioning estimation are analysed in depth and compared with ground truth measurements. Particular attention is given to the UWB positioning system and its behaviour with respect to the environment. The proposed data fusion algorithm provides an overall horizontal and 3D accuracy of 35 cm and 45 cm, respectively, obtained considering 5 different measurement campaigns.

## 1. Introduction

Nowadays, location-based services (LBS) are becoming increasingly important as people need to move and live in complex spaces, usually indoors, where reaching a destination or knowing its position over time allows them to access numerous services, both for commercial and ludic points of view, which deliver context-dependent information. This market is constantly growing and offers innovative tools to support essential services, such as situational awareness, emergency management, healthcare, autonomous navigation and intelligent transport systems, search and rescue, monitoring and management [1].

One of the main reasons for the growth of location-based services is to be found in the worldwide spread of mobile devices, in particular smartphones and tablets, which today are powerful computational tools rich in sensors developed for data transmission and positioning [2,3]. Owning a smartphone allows the individual user to access a multisensory positioning platform, consisting of global navigation satellite systems (GNSS) receivers, inertial platforms, imaging sensors, radio frequency receivers, database of maps and geographic information applications. In addition, the miniaturization of the System-on-chips (SoCs), made up of processors and sensors, has also made it possible to develop multi-sensor platforms even in wearable devices that can be integrated into clothing [4].

Personal tracking devices represent a growing market niche and will gain prominence, as technological advancements will enable devices to hit the mass market and to increase the competitiveness of available solutions. In the context of LBS, personal locator beacons (PLBs) assist rescue authorities in their search to locate people in distress, such as hikers and other adventurers on land and employees working in remote areas.

When LBS are developed for essential services as the one previous described, they need to access and rely on accurate positioning and navigation information, provided continuously both in time and in space, or rather, which allows the location of a body in the transition between outdoors spaces and indoor environments to be estimated continuously assuring accuracy, availability, continuity, reliability and integrity at different levels in function of the application requirements.

Outdoor, the GNSS is the predominant positioning technology, widely used in open-sky condition with well-established performances and real time positioning capability [5]. Unfortunately, satellite-based positioning degrades in a situation where the signal is attenuated by obstacles or affected by interference, like in urban canyons and densely populated areas rich in anthropogenic interferences. To solve these problems, GNSS is often hybridized with inertial sensors, a complementary technology able to sense the movement of a body as accelerations and angular rotation rate and to apply kinematic relation to obtain position, velocity and attitude information [6]. The advantages of hybridizing GNSS with an inertial navigation system (INS) platform are that GNSS is characterized by long-term accuracy which compensate the fast error drift of the INS, while INS are immune from external radio-frequency (RF) perturbation, which compensate the susceptibility of GNSS to interferences. Moreover, the higher positioning update rate of INS allows the continuity of the solution estimation also in GNSS short outage events. By contrast, for long periods of missing GNSS, typical of indoor and underground environments, such hybridization is not sufficient to guarantee positioning, therefore alternative solutions must be used.

In the field of indoor positioning systems (IPS), a plethora of technologies and methods have been investigated relying on cameras [7,8], infrared (Kinect), ultrasound [9], Wireless Local Area Network (WLAN)or Wi-Fi [10,11], mobile communication [12] and more. In man-made environment, any wireless technology can be used for locating and pedestrian tracking can take advantages of existing infrastructure. The Wi-Fi positioning system and Bluetooth low energy (BLE)-based system for example, measure the received signal strength (RSS) and applies multilateration techniques or fingerprinting. They have advantages and limitations over other location technologies, although the more problematic issue is to achieve an accuracy above the room level.

Among them, ultra-wideband (UWB) systems are technologies that uses impulse of radio frequency carrier-less signals to perform localization tasks. UWB are very popular indoor positioning and tracking systems due to their low-cost implementation and the characteristics of the signal which gives major advantages with respect to other radio-frequency based localization techniques. In particular, as the name says, the UWB signal has a wide bandwidth which correspond to a narrow signal in transmission and consequently to an accurate timing capability. Calculating signal round trip time accurately means also precise range measurements and therefore high positioning performance. The miniaturization of these transceivers and their manufacturing low-cost has permitted to install such sensor within mass-market devices. In this regard, Apple was the first to insert a UWB sensor into the latest generation of smartphones although not for positioning tasks [13]. This novelty has attracted considerably the attention of researcher in using UWB as a primary technology in multi-sensor platforms.

Any LBS providing localization for essential tasks like search and rescue or emergency management, must operate in open-sky, in large building, in underground structures or in infrastructure-free spaces where the possibility to localize and track objects and people with standard infrastructure is compromised.

In this context, the main issue of a positioning system is to ensure the ubiquity of the navigation solution, which could be reached only by means of a hybridization procedure based on a multi-sensor platform and data-fusion algorithms.

This paper discusses the architecture of a low-cost multi-sensor platform in which a UWB indoor positioning system is integrated seamlessly with a classical GNSS-INS coupling in order to acquire synchronized data for further data fusion analysis. The present paper is an extension of the conference paper presented in [14]. These data can be used in real time and also in post-processing to evaluate the performance of the system in term of positioning accuracy. In particular, the proposed algorithm aims to solve a navigation task of a pedestrian user moving from an outdoor space to an indoor environment. In an indoor scenario, the UWB-base positioning is the main technology used in this work. Similar to the GNSS, UWB provides ranges between a receiver and several anchors deployed in the environment. These measurements allow them to estimate the position, which can be increased in term of accuracy using the inertial data acquired by an Inertial Measurement Unit (IMU). Outdoor, the GNSS is the primary technology to perform pedestrian navigation. The four main satellite constellations (GPS, Galileo, GLONASS and Beidou) guarantee enough satellite visibility worldwide, ensuring any receiver on the Earth surface to acquire the GNSS signal and therefore to estimate their own position with classical positioning methods and approaches. The quality of the observations (i.e., pseudoranges and carrier-phase measurements) between the receiver and the satellite, together with several estimation techniques, bias modelling and data fusion algorithms, allow nowadays an accuracy of less than 1 m also to be reached with very low-cost receivers and antennas. In this research, a very low-cost GNSS receiver has been used to acquire both raw data and positioning solutions; therefore, as the goal of the research is to perform accurate positioning and navigation, the observation bias must be subtracted using the correction provided by a network of fixed geodetic receiver located on the local territory. Furthermore, in order to further improve positioning, the inertial data acquired during pedestrian motion can be integrated with the GNSS data in a loose coupling integration, typical in kinematic positioning.

The data fusion algorithm exploits the similarity between GNSS and UWB to perform a state estimation based on a loosely coupling architecture and a geofence trigger for switching between indoor spaces and the outdoor environment. The GNSS receiver used is a single-frequency, multi-constellation u-blox Neo M8T [15] while the UWB system is the Pozyx accurate positioning system with an integrated Micro Electro-Mechanical IMU (MEMS IMU) [16]. The main contributions of this study are the following:

- the integration of different low-cost commercial sensor in an integrated multi-sensor platform, able to manage the data stream from different sources, their storage and their use in real time positioning estimation algorithm;
- the development of an algorithm of seamless navigation both in term of continuity of the solution provided, thanks to a geofence approach, and in term of coordinate consistence between outdoor and indoor positioning module;
- the quantitative and qualitative evaluation of the performance of the proposed method in comparison with stand-alone solutions.

The remainder of this paper is organized as follows. Section 2 introduces a review of works related to UWB hybridization for positioning. In Section 3 the Material and Methods used to perform the seamless navigation are provided. The theoretical approach of the present work is described together with the hardware and software used to acquire real-time data. Section 4 describes the experimental setup, the georeferencing procedure of the test area and the acquisition of the reference solution. Section 5 analyses in depth the UWB measurements acquired during the tests, in particular the ranges between the fixed anchors and the moving tag. In Section 6 the results of the positioning estimation and its validation is discussed. Finally, Section 7 presents the conclusions and future work.

## 2. Related Work

Numerous works have focused on the use of UWB and GNSS for pedestrian positioning and navigation. In particular, with regard to UWB, most of the efforts in recent years have focused on characterizing their performance both indoors and outdoors and also in complex environments [17,18]. These analyzes have shown that, despite the excellent performance of these systems for indoor positioning, there are however numerous difficulties mainly related to measurement errors related to non-line of sight (NLOS) and multipath conditions. For this reason, many papers have focused on the characterization of measurement errors and on the search for filtering techniques and outlier rejection [19,20,21] As for the use of UWBs in conjunction with other technologies, little has been done.

Yao et al. [22] describe in their paper an IPS that fuses an UWB positioning system with an IMU sensor-based solution through an ad-hoc extended Kalman filter (EKF) design which coupled tightly the inertial sensors with the measured ranged from the UWB system. Although mainly tested in a simulation environment and designed for 2-dimensional positioning, their proposal demonstrates a significant increase in positioning performances of the hybrid solution with respect to the stand-alone counterpart.

Krukar et al. [23] present an experimental system which fuse two RF-based solution, an UWB system for accurate 3D positioning and a wireless sensor network (WSN) system that enlarge the coverage areas of the UWB. In this case, the UWB positioning is performed with the time difference of arrival technique (TDOA) while the WSN rely on the weighted centroid localization algorithm in the received signal straight indication (RSSI) value. In this case the 2D accuracy move from 4 m, when the receiver is outside the UWB network, to 10 cm when entered in the UWB subsystem range.

Tan and Law [24] formulate an integration between GNSS and UWB in a particular condition, where both ranges from UWB and from satellites are partially visible, as in the case of an indoor space with windows. Even in this case the aims is to verify the improvement in position estimation accuracy of the integrated solution.

Kok et al. [25] combine UWB measurements with inertial measurements to estimate the 6-D pose of a sensor again with a tightly coupled sensor fusion. In this work the major effort was in modelling the UWB measurement errors as a heavy-tailed Cauchy distribution in order to consider NLOS conditions and multipaths. The proposed algorithm reaches centimeter level of accuracy also with data containing a fairly large amount of outliers.

Cebrian et al. [26] analyze the use of UWB-based distance measurements and INS, and provide a hybrid GNSS/UWB/INS sensor fusion strategy for robust seamless indoor/outdoor unmanned aerial vehicle (UAV) navigation while Navarro et al. [27] propose a low-cost multi-sensor fusion positioning prototype which integrates GNSS, INS and UWB measurements to test real-time positioning and communication capabilities in assisted driving applications. In these works, several approaches has been applied for data fusion and sensor integration. For example, in the AGAVE (AGV nAvigation system based on flexible and innovatiVE UWB positioning) project [28] statistical error correction and data fusion technique based on Monte Carlo particle filter has been developed in order to integrate different input (odometry, Differential GPS, UWB, gyroscope for bearing and attitude determination) and provide an accurate position estimation of automatic guided vehicles (AGC).

Most of the previous works were done in indoor controlled environment. According to our best knowledge, few studies were done applying designed estimation algorithms in seamless conditions, with data acquired continuously during the motion of a pedestrian user from an outdoor space to an indoor environment. Moreover, the performance validations of previous works are mainly computed comparing the estimated solution with a reference local grid which may not reflect the real accuracy of the systems. In this work a topographic total station with an angular accuracy of 1” and a distance accuracy of 1 mm + 1.5 ppm has been used in order to provide a real accurate tracking of the user movements. Finally, an interesting aspect considered in our work is the ubiquity of the solution provided in terms of reference coordinate system of the positioning solution. Having a georeferenced UWB test-bed, the transition between GNSS-based solution and UWB-based solution does not require any intermediate module for performing coordinate conversion and can rely simply on the geo-fencing approach.

## 3. Material and Methods

In this section, the hardware and software used for the data acquisition, together with the methodology to perform seamless pedestrian navigation is described.

### 3.1. Hardware

**Figure 1:**
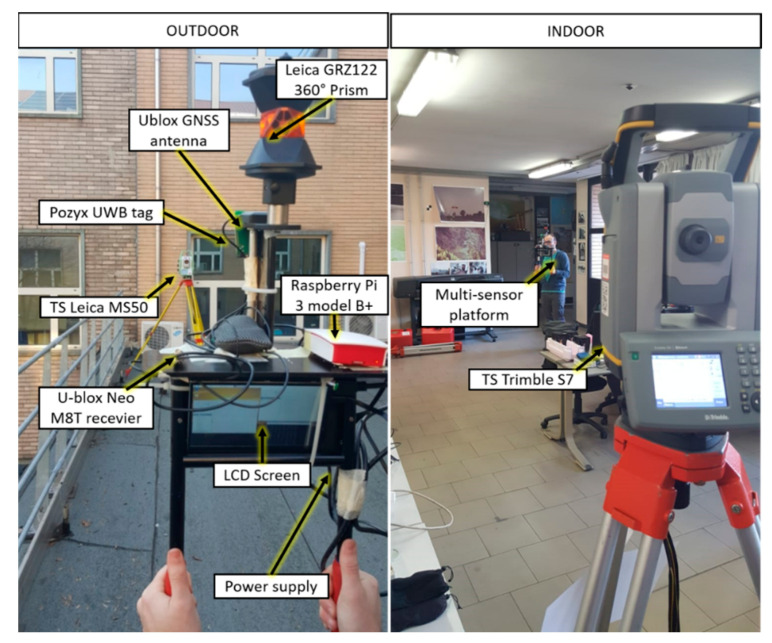
The multi-sensor platform used during the data acquisition campaign tracked by two total stations [14].

The data acquisition and navigation process in this work was performed by a single portable platform for pedestrian navigation, composed of various sensors selected for their complementarity in performing indoor/outdoor navigation (Figure 1). The variety and number of the acquired information allows us to investigate and apply different integration methodologies and algorithms to carry out the task of seamless navigation. The core of the multi-sensor platform is the Pozyx accurate positioning system (Pozyx NV, Ghent, Belgium), a UWB-based real-time locating system (RTLS) based on two-way ranging techniques [29]. The hardware is composed by anchor nodes and tag nodes which both use about 180 mA at max update rate. The inertial acquisitions are demanded to an IMU composed by a three-axis accelerometer, three axis gyroscope and three axis magnetometer and also a micro-barometer integrated on the Pozyx rover board. The GNSS module is the u-blox NEO-M8T (u-blox, Thalwil, Switzerland), a single frequency, multi-constellation receiver developed for automotive application. The computational load of the positioning estimation algorithm is delegated to a Raspberry Pi 3 Model B+ with Ubuntu OS installed board which manage also the serial communication and the time synchronization of all the sensors. The main characteristic of this system is the very low cost of the sensor which nowadays can be easily found also in new generation smartphones. The total cost of the UWB-based multisensory system is about 600€ comprehensive of the anchors network installation. Table 1 summarize the principal characteristics of these sensors together with their performance and cost.

To assess the accuracy of the estimation algorithm a reference solution is required. When a kinematic test is conducted, the irregular path of the pedestrian must be tracked with an accuracy much higher than the accuracy of the device to be validated. In this work, the ground truth was acquired with a topographic total station with the ability to autonomously lock to and track a prism target. In particular, two total stations have been used in order to cover both the indoor and the outdoor part of the acquisition (the Leica MS50 and the Trimble S7 tracking a Leica GRZ122 360 degrees prism).

### 3.2. Methods

The navigation state estimation of the proposed algorithm is based on an EKF and is composed by position (latitude, longitude, altitude), velocity vectors along the north, east and down axes, attitude angles (roll, pitch, heading), acceleration and gyroscopic biases. The filter is used in a feedback form so that when a new measurement is available from a sensor, the error is computed using the Kalman filter which is then used to correct the inertial sensor measurements and navigation parameters.

The EKF is composed by a set of equations applied in two steps recursively: prediction (time update or propagation) and update (measurement aiding or correction). These two steps are based on two estimation models: the state transition model and the measurement model. The state transition model describes the temporal behavior of the states over time and it is used to predict the state vector starting from a previous estimation, whereas the measurement model describes how observations relate to the states being estimated and is used to correct the state vector.

State transition model:

(1) $$x_{k|k-1}=f\left(x_{k-1|k-1}\right)+w_{k}$$

Measurement model:

(2) $$y_{k|k}=h\left(x_{k|k-1}\right)+n_{k}$$

where $w_{k}$ is the state noise vector which follows a normal distribution N(0,Q) and $n_{k}$ is the measurement noise vector follows a normal distribution N(0,R). Finally f(x) and h(x) are two differentiable non-linear functions.

During the prediction phase, the state vector is estimated starting from a previous estimation, through the equations of motion. In the case of an GNSS/UWB-INS integration, the estimate should be represented by the time evolution of position, velocity and attitude ($\dot{p}\;\;\;\dot{v}\;\;\;{\dot{\psi }}_{nb}$) of the inertial platform using the mechanization equations as representation of the dynamic of the system. Since these equations are not linear, a linearization of the mechanization equations is required. In particular, the set of equations which represent the time evolution of the INS error ($\delta \dot{p}\;\;\;\delta \dot{v}\;\;\;\;\delta {\dot{\psi }}_{nb}$) are linear and, therefore, are used to represent the state transition model for GNSS/UWB-INS integration $f\left(\delta {\hat{x}}_{k-1|k-1}\right)$ and consequentially to create the state transition matrix $F_{k}$ of the INS errors. The error propagation can be computed applying a first-order Taylor series expansion to the equation of motion parameterized in the northeast-down (NED) navigation frame and can be simplified in the following form [30]:

(3) $$\delta \dot{p}=T^{\prime }\delta p^{n}+T\delta v^{n}$$

(4) δv˙n=[(Cbnfb) ^]δψnbn+Cbnδfb−[2 (ωien+ωenn) ^]δvn−[(2δωien+δωenn) ^]vn+δgn

(5) δψ˙nbn≈ − [(ωinn) ^ )]δψnbn+δωinn− Cbnδωibb

where the matrix T relates the relates the position errors to their time derivatives, $C_{b}^{n}$ is the rotation matrix, $\delta f^{b}$ and $\delta \omega_{ib}^{b}$ are the accelerometer and rate gyro output errors and $\delta \psi_{nb}^{n}$ is the attitude errors resolved about the NED frame.

Therefore, starting from an initial estimation $\delta {\hat{x}}_{k-1|k-1}$(associated with its var-covariance matrix $P_{k-1|k-1})$, a predicted estimation $\delta {\hat{x}}_{k|k-1}$(with a computed state var-covariance matrix $P_{k|k-1})$ is obtained by applying the state transition model:

(6) $$\delta {\hat{x}}_{k|k-1}=f\left(\delta {\hat{x}}_{k-1|k-1}\right)+w_{k}.\;$$

(7) $$\delta {{\hat{x}}_{k|k-1}}^{T}=F\left(\delta {\hat{x}}_{k-1|k-1}\right)+Q_{k}.\;$$

with the state vector composed by the INS state errors (position p, velocity v, attitude ψ, acceleration and angular velocity biases $b_{a}$ and $b_{g}$). Thus, it can be defined as:

(8) $$\delta x^{T}=\;\left[{\left(\delta p\right)}^{T}\;\;\;{\left(\delta v\right)}^{T}\;\;\;{\left(\delta \psi_{nb}\right)}^{T}\;\;\;{\left(b_{a}^{b}\right)}^{T}\;\;\;{\left(b_{g}^{b}\right)}^{T}\right]\;\;\;\left(15\times 15\right)$$

and the state noise vector $w_{k}$, represented by its var-covariance matrix $Q_{k}$, can be defined as:

(9) $${w_{k}}^{T}={\left[0_{1\times 3}\;\;\;{\left(C_{b}^{n}w_{a}\right)}^{T}\;\;\;{\left(C_{b}^{n}w_{g}\right)}^{T}\;\;\;{\left(C_{b}^{n}\mu_{a}\right)}^{T}\;\;\;{\left(C_{b}^{n}\mu_{g}\right)}^{T}\right]}^{T}\;\;\;\left(15\times 15\right)$$

with $C_{b}^{n}$ rotation matrix and parameter of ${w_{k}}^{T}$ as defined in [31]. Obtaining the state noise covariance matrix $Q_{k}$ and the state transition matrix $F_{k}$, it is possible to compute the state error covariance prediction $P_{k|k-1}$:

(10) $$P_{k|k-1}=F_{k}P_{k-1|k-1}{F_{k}}^{T}+Q_{k}\;\;\;\;\;\left(15\times 15\right)$$

In a closed loop configuration, at each time of INS integration, the state vector is null and is not propagated forward in time.

At the time of update, thus when anindependent solution from the GNSS or UWB system is available, we have the new measurements which are used to correct the predicted solution $\delta {\hat{x}}_{k|k-1}$. The EKF measurement model for GNSS/UWB-INS integration is:

(11) $$\delta y_{k}=H_{k}\delta x_{k}+v_{k}$$

In the loosely coupled integration, this measurements vector is formed as the difference between GNSS/UWB and INS position and velocity ($\delta y_{k}=\;\delta x_{INS}-\delta x_{GNSS/UwB}$) and the H matrix, that relates the observations to the state vector $\delta x_{k}$, can be defined as:

(12) $$H=\left[I_{6\;\times 6}\;\;\;\;\;0_{6\;\times 9}\right]$$

Finally, it is possible to estimate the measurement noise vector $v_{k}$, represented by its var-covariance matrix $R_{k}$ defined a priori.

This measurement model is used to update the state vector and its var-covariance matrix. Therefore, the Kalman gain matrix is required and can be estimated using the following equation:

(13) $$K_{k}=\;P_{k|k-1}H_{k}^{T}{\left(H_{k}P_{k|k-1}H_{k}^{T}+R_{k}\right)}^{-1}$$

Finally, the pre-GNSS/UWB measurement estimates of the state error $\delta x_{k}$ and the correspondent $P_{k|k}$ matrix are refined with equation:

(14) $$\delta {\hat{x}}_{k|k}=K_{k}\delta y_{k}$$

(15) $$P_{k|k}=\left(I-K_{k}H_{k}\right)P_{k|k-1}$$

Then, the INS parameters are corrected for the estimated state error vector:

(16) $${\hat{x}}_{k|k}={\hat{x}}_{k|k-1}+\delta {\hat{x}}_{k|k}$$

The GNSS receiver and the Pozyx GNSS inertial sensor unit are the systems integrated in a loosely coupled algorithm that represents the starting navigation solution for the outdoor space. The GNSS receiver provide position at 1 Hz, while the IMU works at 4 Hz. This high rate is fundamental to fill the gap between two subsequent GNSS measurements. The GNSS position and velocity are used to estimate the INS error. When the systems move indoor the GNSS receiver is no more able to provide measurements so it has been substituted by the UWB receiver that enters in the UWB network of fixed anchor nodes. Also, the UWB can work at 1 Hz providing positioning in the indoor environment. The previous loosely coupled integration is used in this case simply using the UWB data to update the INS navigation.

In the proposed work the 3D localization problem of the UWB positioning is solved with an iterative non-linear least square estimation. This method is applied iteratively in order to refine the linearization point which is obtained when the error *r* is lower than the preset tolerance. If *m* is the number of anchors in the network, the *i*_th_ residuals can be written as a function of the coordinates of the target node:

(17) $$r_{i}=d_{i}-\sqrt{{\left(x-x_{i}\right)}^{2}+{\left(y-y_{i}\right)}^{2}+{\left(z-z_{i}\right)}^{2}}$$

with $d_{i}$ the distance between two nodes, ${\left(x,y,z\right)}^{T}$ the coordinates of the target node and ${\left(x_{i},y_{i},z_{i}\right)}^{T}$ the known position of the *i*_th_ anchor. At each step of the iteration, starting from an initial guess $x_{0}$ for the position of the target node, the algorithm returns a value $x_{k+1}$ that minimizes the sum of the residuals *S*:

(18) $$S=\overset{m}{\underset{i=1}{\sum }}{r_{i}}^{2}$$

the estimation value is given by:

(19) $$x_{k+1}=x_{k}-{\left(J^{T}J\right)}^{-1}J^{T}r\left(x_{k}\right)$$

where ***J*** is the Jacobian matrix of the residual vector function.

## 4. Test Setup

**Figure 2:**
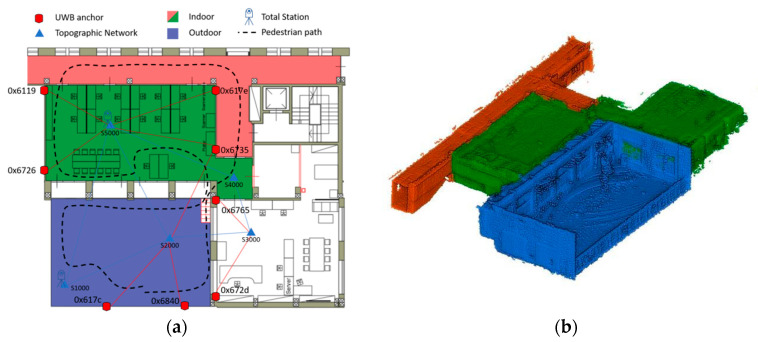
(**a**) Graphical representation of the test with the pedestrian path moving from an outdoor space to an indoor environment. The topographic network, the ultra-wideband (UWB) anchors location and the total stations position are also shown. (**b**) 3D visualization of the outdoor (blue) and indoor (green + red) environments.

The analysis according to the above methodology was performed on measurements acquired by a multi-sensor platform in continuous acquisition during a pedestrian motion. In order to acquire representative data of seamless navigation, the pedestrian user carrying the platform has followed a path that, starting from an open environment, develops towards an indoor space, and then returns outdoors. The data acquisition was performed in January 2020 in the geomatics laboratory of the Politecnico di Torino (Italy, Latitude45.063332°, Longitude 7.660458° considering the WGS84 reference system with the UTM32N projection), an experimental laboratory that overlooks an outdoor terrace. The trajectory trave1led, in addition to presenting several changes of direction, also presents a variation in level in conjunction with the passage from the terrace to the laboratory (Figure 2).

### 4.1. Ground Truth

In order to perform a validation in term of positioning accuracy of the estimated solution, a reference trajectory provided by direct observation is required. This trajectory, used as ground truth, must be of greater accuracy than the solution to be validated and, moreover, must be simultaneously acquired. Therefore, two total stations (the Leica MS50 of Leica Geosystems AG, Heerbrugg, Switzerland and the Trimble S7 of Trimble Geospatial, Sunnyvale, CA, USA) located on the vertices of a small georeferenced topographic network, autonomously locked and traced a moving 360° prism target (Leica GRZ122), mounted on the multi-sensor platform. Having georeferenced the network, the components of the position of the prism also appear to be in the same reference frame. The georeferencing of this network took place by locating two dual-frequency multi-constellation geodetic GNSS receivers (Leica GS14 and GS18), in static acquisition for several hours on the materialized vertices. The observations were subsequently post-processed with a differential approach and the network compensated in order to obtain the accurate coordinates of the points (cm-level accuracy) projected in a cartographic system (WGS84 reference system with the UTM32N projection). Table 2 shows the coordinates obtained.

### 4.2. Ultra-Wideband (UWB) System Setup

In order to employ the UWB technology for positioning, different algorithms and estimation procedure have been developed which relies on measurements based on the radio signal traveling between the fixed nodes and target nodes in addition to the position information of the fixed nodes.

The Pozyx system used in this work provides high-precision distance measurement between a network of fixed sensors (anchors) placed on the edge of the system’s operating area and a mobile sensor (tag) using a two-way ranging approach. In particular, 8 anchors were placed around the testing area and their position was calculated with high accuracy through detailed measurements (angles and distances) made with a total station. As previously described, the total station was placed on a known point belonging to the topographic network in order to trace the local system back to a global georeferenced system. Consequently, any estimation algorithm used for positioning (least-square, multilateration, Kalman filtering, etc.) provides the tag position in the absolute reference frame that in this work correspond to the WGS84 reference system with the UTM32N projection.

### 4.3. Geofencing

Having the UWB position estimation expressed in a geographic reference system allows a data-fusion algorithm to be developed which easily combines the GNSS observations and provides coordinate consistency between outdoor and indoor positioning module. Another major advantage of working with georeferencing measurements is that the observations are related to a well-defined space with well-known boundaries. This means that the estimated position, and also the raw measurements, are not related to a virtual local reference frame any more but are connected with a geographic zone and therefore acquire spatial awareness. This condition allows a geofencing approach to be used in the proposed estimation framework which consist in defining a virtual static perimeter which boundaries are expressed in geographic coordinates. As tracked mobile objects move across the geofence, an algorithm switch may be triggered. In our work, two polygons were defined, one corresponding to the georeferenced area of the indoor laboratory and another corresponding to an adjacent indoor corridor. The trigger between two estimation algorithms is performed only when that used moves from the outdoor environment to one of the two geofence. 

### 4.4. Data Acquisition

The data acquisition was performed assembling all the sensor in a handled platform together with an LCD screen for real time error checking and a prism mounted on the top. Each lever-arm between the sensors (GNSS antenna, UWB antenna, 360° prism) was measured with a caliber. The positioning algorithm was performed in real-time through the Raspberry Pi processor. The data acquisition, the communication protocols and the processing were undertaken all in the same Python environment.

**Figure 3:**
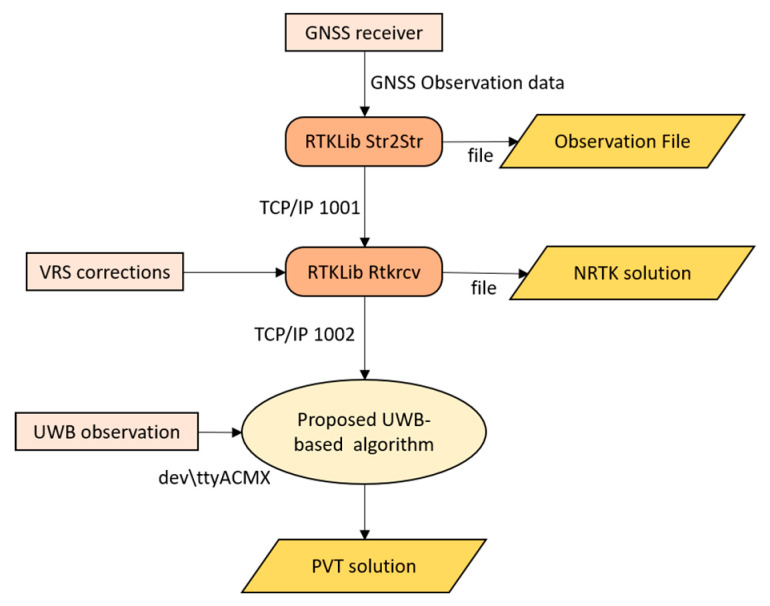
Communication protocols and signal flow management [14].

A major issue was to provide to the EKF the GNSS state vector already processed in a network real-time kinematic (NRTK) positioning mode. To do this, the u-blox receiver has been connected through USB port to the Raspberry board where a modified version of the RTKLIB 2.4.3 has been compiled. Here, through the *str2str* module the GNSS signal coming from the antenna is redirected on a Raspberry port through TCP/IP protocol. This flow is then split in order to allow both real-time and post-processing positioning: one data flow is saved as .ubx file for the quality control and eventually for post-processing positioning while the same flow split into another port has been used by the *rtkrcv* for the NRTK positioning. This module uses this flow as rover input and the differential corrections obtained from the SPIN (Servizio di Posizionamento Interregionale GNSS) network of permanent stations for performing the real-time positioning, considering the virtual reference station (VRS) correction. As demonstrated in [3,5], this positioning method allows the achievement of centimetric precision and accuracy in real-time. Finally, this solution is used as input for the integrated positioning algorithm presented in this work. The same flow acquired by the TCP/IP port is also saved for a quality analysis and for the possibility of post-processing the data with other techniques (Figure 3). The presence of raw GNSS observations of code and phase allows the implementation of tightly coupled approaches subject of future work.

## 5. Evaluation of UWB Data

The GNSS stand-alone positioning error is around 8 m with a 95% of probability. The real-time positioning error obtained applying differential corrections broadcasted by a network of permanent station is about 37 cm ± 21 cm in planimetry. Assessing the positioning performances of GNSS is a widely analyzed topic in scientific literature, while evaluating the behavior of a UWB positioning system in term of acquired observation and positioning capability in complex environment remains interesting. First of all, the data acquired by the UWB during the measurement campaign both indoors and outdoors has been considered. The aim is to observe the behavior and consequently the performances of a UWB positioning and tracking system in a real complex scenario, where a pedestrian user moves between different environments that contains objects and people. The outdoor-to-indoor path results in a no-line of sight condition for the tag and, therefore, in various propagation patterns, including shadowing, reflections and scattering. Assessing the performances of the UWB-only solution related with the user neighborhood, allows the benefits of the multi-sensor hybridization to be enhanced and provides qualitative and quantitative evidence useful to tuning data-fusion algorithms.

**Figure 4:**
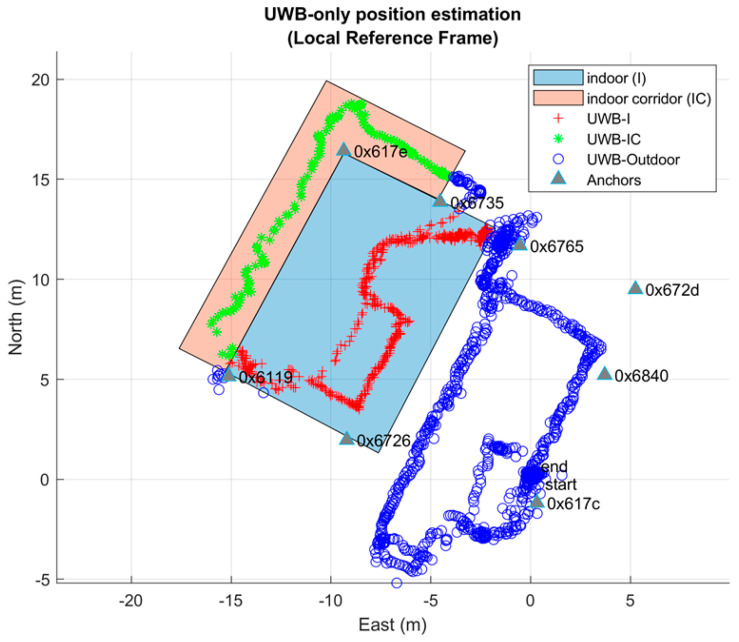
The horizontal positioning estimation obtained by the UWB only in real time.

Firstly, Figure 4 reports the positioning results of the UWB system obtained in real time during the first test of pedestrian walking. The eight anchors position and the relative identification code are plotted with a triangle mark. Two colored polygons represent two different types of indoor environment. The cyan-colored rectangle represents the laboratory area, which falls completely within the network of anchors. The pink polygon, on the other hand, represents an internal corridor adjacent to the laboratory totally outside the anchors network. The remaining space represents the outdoor environment. Observing this figure it is evident that outdoor, almost the entire path is surrounded by anchors even if only two anchors are in LOS (anchor 0x617c and 0x6840) while the others are placed on the internal surface of the perimeter walls of the building and consequently are shielded. When the user walks inside, at least 4 anchors are visible simultaneously until he exits the corridor, a condition in which no anchors are in LOS. Therefore, despite the visibility of at least 4 anchors is not guaranteed for most of the route, the plot shows a continuous and not-interrupted 2D position estimation for the entire path (blue, red and green marks). This confirms the UWB’s high material penetration properties. Regarding the measured accuracy of this solution, more details will be provided in Section 6, Results, in comparison with our seamless solution.

### 5.1. Visibility Analysis

**Figure 5:**
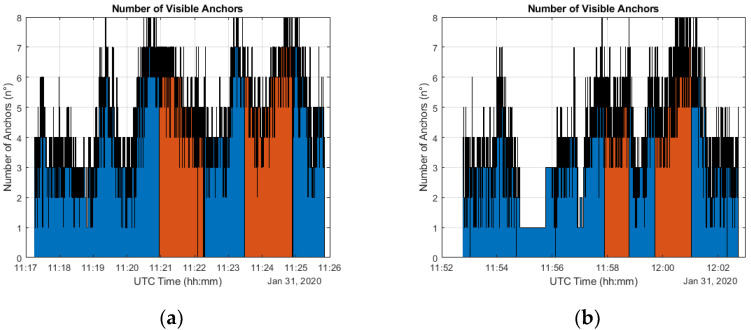

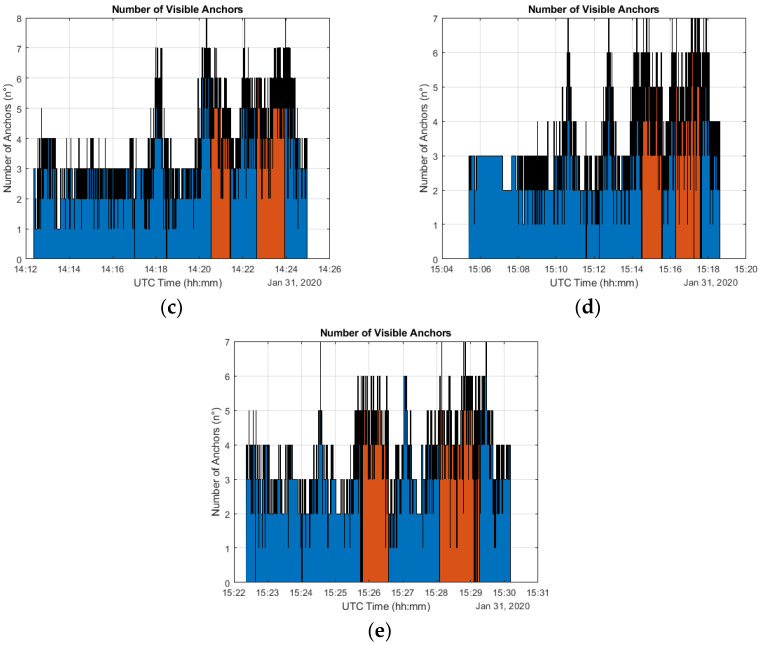
Number of measured ranges by the moving tag during the acquisition time for five different tests (**a**–**e**). Ranges are both in LOS and NLOS conditions.

To analyze more in deep the UWB’s high material penetration property, an anchor visibility analysis is performed for all five test and reported in Figure 5, where the number of visible anchors is plotted in function of the time. In this case, the visibility consists in both LOS and NLOS received signals and, therefore, is representative of the number of ranges (also biased) used in the position estimation. Thanks to the time reference it is possible to identify the portion of the plot which correspond to the indoor portion of the path represented in red. As supposed, the majority of contemporary ranges acquisition is concentrated in this area with a variation of visible satellites between 4 and 8. Moreover, there are also other areas which present a high number of signals received simultaneously, once again to indicate the penetrative properties of the UWB signal.

Nevertheless, the visibility plot shows the lowest number of acquired ranges in the first figure section, which correspond to the outdoor environment. This consideration is important as it provides the first motivation for relying on GNSS positioning in open-sky conditions.

### 5.2. Analysis of Range Measurements

**Figure 6:**
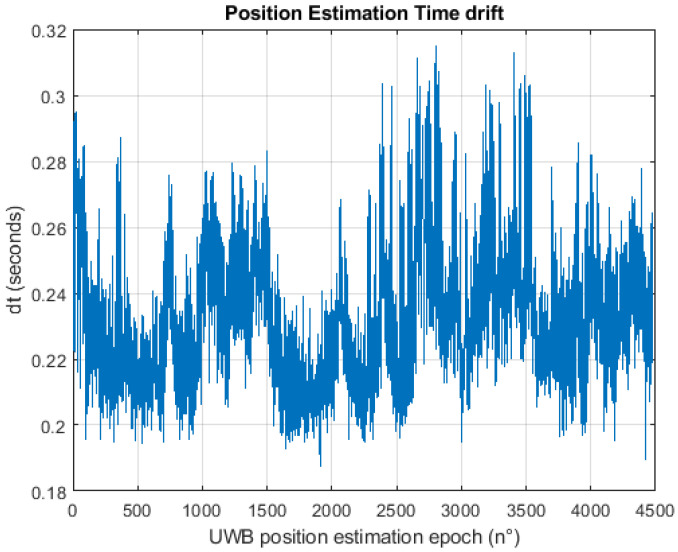
Position estimation time drift.

As previously stated, during the measurement campaign, the data acquisition and the processing were made all in the same python environment allowing both raw data and positioning estimation to be stored. Considering only the Pozyx UWB system, several data were acquired and stored like the ranges between the tag and the anchors network, the received signal straight RSS, the inertial measurements (acceleration, rotation rate, magnetic force) and finally the position estimation from our algorithm with the relative timestamp. The positioning update rate achieved by the Pozyx system depends on several parameters like the length of the preambles, the bitrate, the number of anchors, the communication protocols and the kind of raw data acquired. The manufacturer states that, with only four anchors and one tag, the best parameters combination allows a positioning update rate of around 140 Hz to be reached. Due to the complexity in characterizing the update rate with such variability, in the present work only the time variation for all the tests performed which have the same parameters and settings is reported. Figure 6 gives a good indication of the delay that can be expected in positioning estimation showing a mean time variation value of 0.24 s with a maximum of 0.34 s and a standard deviation of 0.03 s. Therefore, it is possible to summarize that the systems update rate is around 4 Hz which is useful for pedestrian navigation purposes and it is also under the update rate of the low-cost GNSS receivers used in this work.

What is important to observe is that in the 0.2 s, the systems acquired in sequence all the data useful to perform the positioning and in particular a maximum of 8 range measurements from the anchors placed around the environment. In order to perform the positioning in real time, the ranges measurement timestamp has been packed (merged) in the positioning update rate therefore we are not able to observe their rate of acquisition.

Thanks to the user continuous total station tracking, a reference ground truth of the walking path was acquired. The obtained data are East, North and Altitude position in the cartographic reference system. Knowing also the position of the anchors in the same reference frame, it is possible to derive the real ranges for all positioning time, thanks to the following geometric range model:

(20) $$R=\sqrt{{\left(E_{anc}^{i}-E_{tag}^{t}\right)}^{2}+{\left(N_{anc}^{i}-N_{tag}^{t}\right)}^{2}+{\left(U_{anc}^{i}-U_{tag}^{t}\right)}^{2}}$$

where:

$E_{anc}^{i}$, $N_{anc}^{i}$, $U_{anc}^{i}$ are the East, North and Vertical components of the i-th anchor.

$E_{tag}^{t}$, $N_{tag}^{t}$, $U_{tag}^{t}$ are the East, North and Vertical components of the moving tag at the time t.

As these ranges are synchronized and aligned with the UWB raw ranges acquired in real time, it is possible to analyze the behavior of the system in all the environment crossed during the path. UWB sensors manufacturers usually declare the ranging capabilities of their system that usually don’t reflects complex scenarios and real case applications. Therefore, computing errors of the range measurements acquired during a kinematic test both indoors and outdoors provide accuracy and precision of the system in a more realistic scenario. The two-way ranging algorithm implemented by host system software Pozyx is the single sided two-way ranging (SS-TWR) which involves the measurement of the round trip time of a message sent from a tag to an anchor and received back after a certain delay and the measurement of the replay time. Both times are measured independently by the two devices involved in the communication using their respective internal clock. These clocks have both have their own clock offset error and clock drift and, therefore, the resulting time of flight estimate has an error that increase as the replay time increase. One should note that the time replay include also the message length which also affect the TOF. In our experience the ultra-wideband was tested with several settings but the results of the present work are obtained using channel 5, preamble length 1024, prf 64 MH, and bitrate of 110 kbps. With these parameters the clock induced error is about 1 ns. The range between all the anchors and the moving tag for all five test has been compared with the geometric range obtained by the TS direct measurements and the statistical parameters of the errors are reported in Table 3**.**

**Figure 7:**
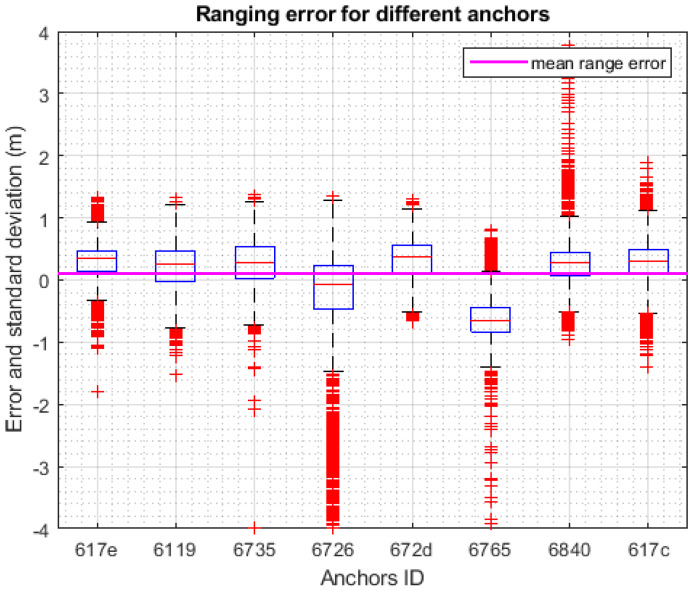
Ranging error of Pozyx UWB system for each anchor.

This allows also the effect of a multipath signal in TWR to be evaluated observing the distance errors. Note that the UWB signal overcomes the multipath effects compared to other narrow-band signals. However, signal disturbance because of multipath effects in UWB is still noticeable in distance error estimation, as shown by the different behavior of anchors 0x6726 and 0x6765 visible also in Figure 7.

In Table 4 the Root Mean Square Error (RMSE) and the % of outlier for each anchor is reported. The outlier is defined as the values belonging to the first and fourth quartiles of the weighted average distribution.

### 5.3. Received Signal Strength (RSS) vs. Ranges

**Figure 8:**
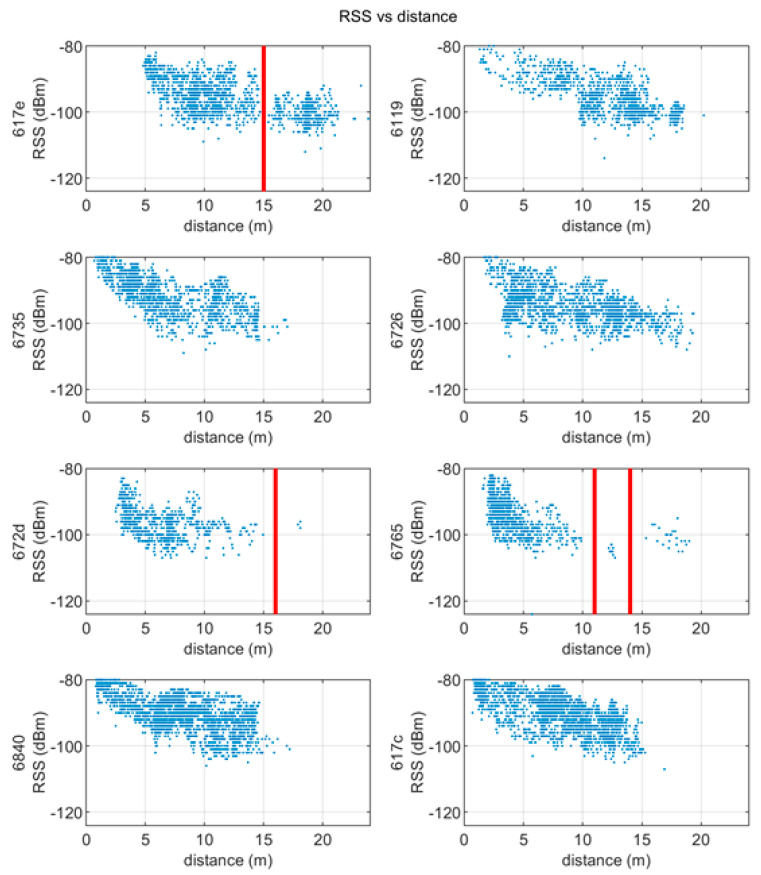
Received signal strength vs. measured distances for each anchor during the pedestrian test.

Another important indicator regarding how the signal is affected by the environment is provided by the received signal strength expressed as power at the receiver tag. According to theoretical formulation, the power increase as the tag approach an anchor while it decreases with increasing distance or in presence of obstacles. This is confirmed from the plots in Figure 8, in which the RSSI is plotted against the tag-anchor distance during the measurement campaign. The plot is repeated for all 8 anchors and considers only the value above −80 dBm which is the threshold below which the system is unable to receive. With the setting defined in the proposed test, −103 dBm is the lowest RSS value at which reception is possible in Pozyx systems.

In some plots (in particular for anchors 0x617e, 0x672d and 0x6765) it is possible to observe an interruption in the point cloud representing the power with respect to the distance. As the path is continuous, these interruptions probably correspond to obstacles through which the signal has not been able to penetrate, probably in conjunction with wardrobes leaning against the walls or particularly thick walls.

## 6. Results

**Figure 9:**
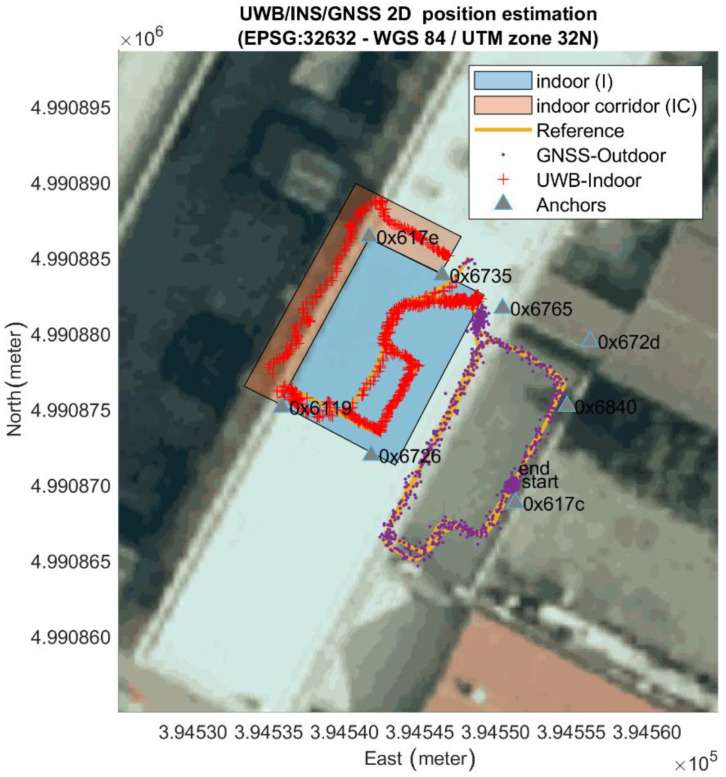
Pedestrian trajectory obtained by the proposed algorithm expressed in WGS84-UTM zone 32 N coordinates.

**Figure 10:**
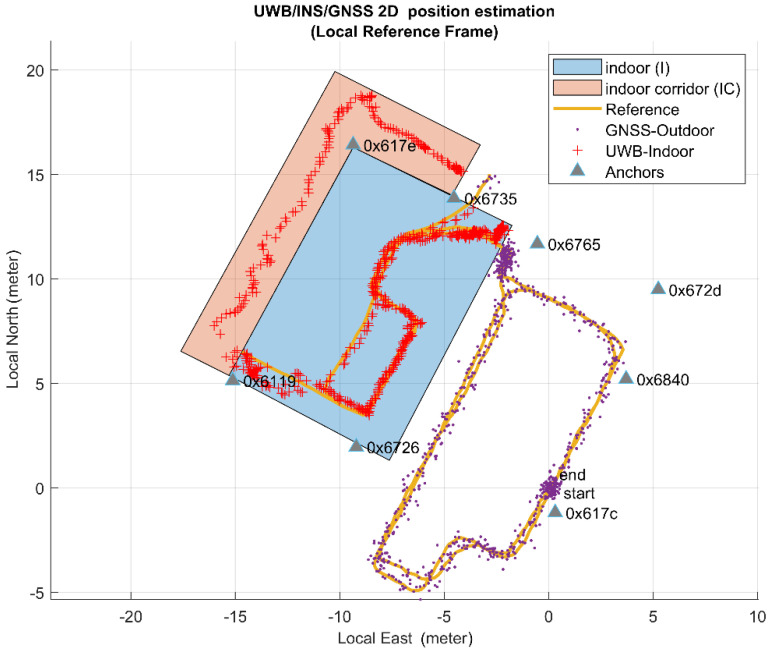
Pedestrian trajectory obtained by the proposed algorithm expressed in local reference system.

To verify the accuracy of the proposed algorithm in performing seamless navigation estimation of pedestrians, we conducted several experiments consisting in a user walking along a random path from the outdoor terrace to the indoor laboratory. The user walked for a short period outside the laboratory and therefore outside the geofence area. This allows also the behavior of the algorithm in no-line-of-sight (NLOS) conditions to be analyzed and with fewer anchors to observe. In fact, the environment has a significant impact on the accuracy of measurements and consequently on the accuracy of the positioning, therefore the real time estimation was performed outdoors, indoors inside the network of UWB, and in a narrow corridor with no LOS anchors. In total, five different acquisition and real-time processing tests were made during the measurement campaign with the aim to evaluate also the reliability of the methodology. The proposed estimation algorithm based on GNSS and UWB data has provided 5 different positioning solution which were compared with the trajectory obtained with the total stations and of which the positioning error along the vertical component, in 2D and in 3D was calculated. Figure 9 shows the obtained 2D trajectory in cartographic coordinates superimposed on the orthoimage from the “Agenzia per le Erogazioni in Agricoltura” AGEA 2018 (pixel resolution 30 × 30 cm) for Test n° 1, where it is possible to notice the outdoor terrace and the indoor lab. Figure 10 shows the same trajectory in local reference frame in a plot grid of 5 m × 5 m. In this image GNSS real-time position is shown as purple dots, the UWB-based solution is shown as red cross and the reference solution is represented by an orange line (data from the total station). It is possible to observe the absence of reference solution in the indoor corridor part of the path due to the presence of the walls. Therefore, the error analysis was performed only between the common part based on common timestamps. For each total station acquisition, the closest UWB estimation time was identified and compared, while intermediate measurements were discarded as the UWB operates at 4 Hz and the TS at 1 Hz. A check on the linearity of the data and on the time difference was performed in order to verify the presence of time delay between the two system. The two data were both aligned with respect to the GPS time.

Let ΔEi, ΔNi and ΔUi the errors in the East, North and Vertical components of the *i*-th position estimate sample. The RMS vertical, horizontal (2-D) and 3-D errors are defined as:

(21) $$\mathrm{RMSE}\;\left(\mathrm{UP}\right)=\sqrt{\frac{1}{n}\sum_{i=1}^{n}\Delta U_{i}^{2}}$$

(22) $$\mathrm{RMSE}\;\left(2D\right)=\sqrt{\frac{1}{n}\sum_{i=1}^{n}\left(\Delta E_{i}^{2}+\Delta N_{i}^{2}\right)}$$

(23) $$\mathrm{RMSE}\;\left(3D\right)=\sqrt{\frac{1}{n}\sum_{i=1}^{n}(\Delta E_{i}^{2}+\Delta N_{i}^{2}+\Delta U_{i}^{2})}$$

Table 5, Table 6 and Table 7 shows the statistical parameters of the positioning estimation errors for horizontal, vertical and 3D coordinates.

The results obtained show similar accuracy in the vertical component with respect to the horizontal one, in contrast with the typical behavior of the trilateration estimation, where the vertical accuracy usually is worse due to the weak geometry. The reason is that in this work some of the UWB anchors where placed under the mean horizontal plane as the terrace level is lower than the laboratory level. Moreover, observing the 3D RMSE of the five tests, whose variation correspond to few centimeters, is possible to affirms that, if the environment is always the same, the solution proposed is reliable in time.

### Comparison between UWB-Only vs. Global Navigation Satellite Systems (GNSS) + UWB

**Figure 11:**
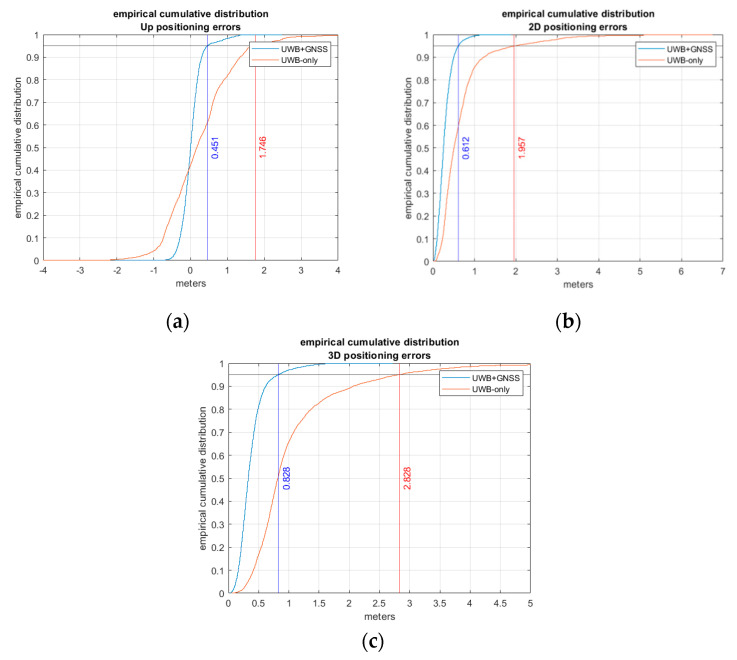
Empirical cumulative distribution of the positioning errors expressed in meters of UWB solution vs. the proposed solution for (**a**) the vertical, (**b**) horizontal and (**c**) 3D component.

Considering the overall positioning error of the five tests obtained by the proposed estimation procedure, the empirical cumulative distribution of the errors was computed and compared with that obtained by the real-time solution provided by the UWB only. From this comparison it is possible to observe that the errors estimated by the UWB-only solution are significantly larger than the method proposed. Figure 11 shown this comparison with also the 95th percentile of the vertical, horizontal and 3D positioning error.

From Table 8, Table 9 and Table 10 it is possible to observe that the mean error and the RMSE of the proposed solution are lower than the corresponding value for the UWB-only real-time solution. The reason is mainly due to the increase in accuracy in the outdoor part of the path by the GNSS positioning.

It is important to underline that, as seen in the Section 5, UWB positioning is guaranteed for the entire test path, both outdoors and outside the anchor network (corridor); therefore, in the case of loss of the GNSS signal, the solution will still be available even if it has deteriorated.

## 7. Conclusions

UWB positioning systems are becoming increasingly important for pedestrian navigation in limited environments, both indoors and outdoors, thanks to the numerous advantages they present both in terms of cost and signal transmission. However, the performance of these systems is strongly affected by the environmental conditions in which they operate. GNSS technology is still the preferred positioning system outdoors, thanks to its integrity and accuracy. By combining these two positioning technologies it allows a seamless solution to be obtained, that is capable of obtaining the continuous positioning both outdoors and indoors. This paper proposes a pedestrian seamless navigation methodology, based on a low-cost multi-sensor platform, a data fusion algorithm that integrates GNSS, UWB and INS, and a context awareness approach based on geofencing. In this way the positioning is georeferenced and no longer tied to the local reference system defined by the network of sensors placed around it. The data fusion algorithm is an EKF applied to the specific system which provides an overall horizontal accuracy of 35 cm obtained analyzing 5 different tests. In 3D the solution provides a positioning accuracy on 45 cm. The paper analyzes also very deeply the raw measurements acquired by the UWB commercial system Pozyx in relation with the test-site environment. The ranging error of the system used in this specific cinematic test was around 30 cm which is in line with the performance analysis conducted by the system manufacturer. This level of error is typical of low-cost UWB systems based on the SS-TWR method such as the system presented. However, the DW1000 UWB chipset provides the facilities for message time-stamping and precise control of message transmission times that enable also more advanced algorithms like double-sided two-way ranging (DS_TWR), which has a reduced error even for quite long response delays. This protocol is not implemented at the moment but could represent a solution for further improving the ranging accuracy. Knowing the behavior of the system in such complex environments (outdoor, indoor, narrow corridor) will provide insights for future research on error rejection.

## Figures and Tables

**Table 1:** Performance specification of multisensor system.

	UWB	IMU	Processing Unit
NEO-M8T Ublox	Pozyx	Pozyx	Raspberry Pi 3 Model b+
Constellation: GPS/GLONASS Galileo/BeiDou	3D position accuracy: 30 cm	3 axes Accelerometer, Gyroscope and Magnetometer	OS: GNU/Linux
2D position error: 2.5 m	Antenna: Decawave DW1000	Roll 2 deg Pitch 5 deg Heading 4 deg	Ports: 4 USB 2.0; 1 Ethernet
Max navigation update rate: 5 Hz	Max Ranging update rate: 140 Hz	Max Update Rate: 100 Hz	RAM: 512 MB
-	Max Positioning update rate: 80 Hz	-	-
-	Typical LOS range: 30 m	-	-

**Table 2:** Coordinates of the geo-referenced topographic network and UWB anchors in UTM-WGS84 32N.

Point	East (m)	North (m)	H Ellips. (m)
TS Leica	394,547.830	499,0864.307	302.566
TS Trimble	394,540.988	499,0878.101	303.492
0x6726	394,541.794	499,0871.963	305.888
0x6119	394,535.877	499,0875.138	305.985
0x617e	394,541.642	499,0886.423	306.157
0x6735	394,546.468	499,0883.866	305.827
0x6765	394,550.462	499,0881.682	305.869
0x672d	394,556.253	499,0879.496	305.554
0x6840	394,554.713	499,0875.212	303.851
0x617c	394,551.317	499,0868.819	303.837

**Table 3:** Statistical parameter of the errors in ranging measurement for 8 anchors during all 5 tests performed.

	0x617e	0x6119	0x6735	0x6726	0x672d	0x6765	0x6840	0x617c
max (m)	1.33	1.33	1.38	1.36	1.31	0.82	3.78	1.89
min (m)	−1.79	−4.31	−5.96	−8.09	−0.66	−4.19	−0.95	−1.40
mean (m)	0.30	0.20	0.25	−0.62	0.34	−0.65	0.28	0.30
std (m)	0.31	0.43	0.52	1.61	0.33	0.50	0.41	0.33

**Table 4:** Ranges RMSE and % of outliers for each anchor.

Anchor	RMSE (m)	Ranges	Outlier	Outlier %
0x617e	0.43	2562	145	6%
0x6119	0.48	1476	48	3%
0x6735	0.58	2043	30	1%
0x6726	1.73	2373	402	17%
0x672d	0.47	1129	18	2%
0x6765	0.82	1346	125	9%
0x6840	0.5	4268	197	5%
0x617c	0.45	4068	134	3%

**Table 5:** Vertical positioning error.

	Test 1	Test 2	Test 3	Test 4	Test 5
max (m)	1.24	2.48	1.48	1.55	1.94
mean (m)	0.05	0.01	0.03	0.00	0.02
RMSE (m)	0.31	0.27	0.31	0.25	0.31

**Table 6:** 2D positioning error.

	Test 1	Test 2	Test 3	Test 4	Test 5
max (m)	1.02	1.26	1.11	1.78	1.87
mean (m)	0.29	0.31	0.27	0.27	0.30
RMSE (m)	0.34	0.37	0.31	0.32	0.37

**Table 7:** 3D Positioning error.

	Test 1	Test 2	Test 3	Test 4	Test 5
max (m)	1.56	2.72	2.09	2.12	2.20
mean (m)	0.39	0.40	0.37	0.35	0.40
RMSE (m)	0.46	0.46	0.44	0.41	0.48

**Table 8:** Overall vertical positioning error.

	UWB-Only	GNSS+UWB
max (m)	9.75	2.48
mean (m)	0.25	0.02
RMSE (m)	1.00	0.29

**Table 9:** Overall horizontal positioning error.

	UWB-Only	GNSS+UWB
max (m)	6.75	1.87
mean (m)	0.69	0.28
RMSE (m)	0.95	0.34

**Table 10:** Overall 3D positioning error.

	UWB-Only	GNSS+UWB
max (m)	12.87	2.72
mean (m)	1.07	0.38
RMSE (m)	1.38	0.45

## References

[1] Kolodziej K.W., Hjelm J. (2017). Local Positioning Systems: LBS Applications and Services.

[2] Linty N., di Pietra V., Dabove P. (2019). Positioning exploiting GNSS raw measurements. Smartphones: Recent Innovations and Applications.

[3] Dabove P., di Pietra V. (2019). Towards high accuracy GNSS real-time positioning with smartphones. Adv. Space Res..

[4] Bousdar Ahmed D., Diaz E.M., García Domínguez J.J. (2020). Automatic Calibration of the Step Length Model of a Pocket INS by Means of a Foot Inertial Sensor. Sensors.

[5] Dabove P., Di Pietra V. (2019). Single-Baseline RTK Positioning Using Dual-Frequency GNSS Receivers Inside Smartphones. Sensors.

[6] Gleason S., Gebre-Egziabher D., Egziabher D.G. (2009). GNSS Applications and Methods.

[7] Anwar Q., Malik A.W., Thörnberg B. Design of coded reference labels for indoor optical navigation using monocular camera. Proceedings of the International Conference on Indoor Positioning and Indoor Navigation, Montbeliard-Belfort.

[8] Deretey E., Ahmed M.T., Marshall J.A., Greenspan M. Visual indoor positioning with a single camera using PnP. Proceedings of the 2015 International Conference on Indoor Positioning and Indoor Navigation (IPIN).

[9] Vidas S., Lakemond R., Denman S., Fookes C., Sridharan S., Wark T. An Exploration of Feature Detector Performance in the Thermal-Infrared Modalit. Proceedings of the 2011 International Conference on Digital Image Computing: Techniques and Applications.

[10] Kim B., Bong W., Kim Y.C. Indoor localization for Wi-Fi devices by cross-monitoring AP and weighted triangulation. Proceedings of the 2011 IEEE Consumer Communications and Networking Conference (CCNC).

[11] Retscher G., Gikas V., Hofer H., Perakis H., Kealy A. (2019). Range validation of UWB and Wi-Fi for integrated indoor positioning. Appl. Geomat..

[12] Shahmansoori A., Uguen B., Destino G., Seco-Granados G., Wymeersch H. (2019). Tracking Position and Orientation Through Millimeter Wave Lens MIMO in 5G Systems. IEEE Signal Process. Lett..

[13] The Biggest iPhone News Is a Tiny New Chip Inside It. https://www.wired.com/story/apple-u1-chip/.

[14] Di Pietra V., Dabove P., Piras M. Seamless Navigation using UWB-based Multisensor System. Proceedings of the 2020 IEEE/ION Position, Location and Navigation Symposium (PLANS).

[15] NEO/LEA-M8T Series. https://www.u-blox.com/en/product/neolea-m8t-series.

[16] Barral V., Suárez-Casal P., Escudero C.J., García-Naya J.A. (2019). Multi-sensor accurate forklift location and tracking simulation in industrial indoor environments. Electronics.

[17] Ridolfi M., Van de Velde S., Steendam H., De Poorter E. (2018). Analysis of the Scalability of UWB Indoor Localization Solutions for High User Densities. Sensors.

[18] Alarifi A., Al-Salman A., Alsaleh M., Alnafessah A., Al-Hadhrami S., Al-Ammar M.A., Al-Khalifa H.S. (2016). Ultra Wideband Indoor Positioning Technologies: Analysis and Recent Advances. Sensors.

[19] Barral V., Escudero C.J., García-Naya J.A., Suárez-Casal P. (2019). Environmental Cross-Validation of NLOS Machine Learning Classification/Mitigation with Low-Cost UWB Positioning Systems. Sensors.

[20] Wymeersch H., Maranò S., Gifford W.M., Win M.Z. (2012). A machine learning approach to ranging error mitigation for UWB localization. IEEE Trans. Commun..

[21] Khodjaev J., Park Y., Malik A.S. (2010). Survey of NLOS identification and error mitigation problems in UWB-based positioning algorithms for dense environments. Ann. Telecommun..

[22] Yao L., Wu Y.A., Yao L., Liao Z.Z. An integrated IMU and UWB sensor based indoor positioning system. Proceedings of the 2017 International Conference on Indoor Positioning and Indoor Navigation (IPIN).

[23] Krukar G., Wenzel M., Karbownik P., Franke N., von der Grün T. Proof-of-concept real time localization system based on the UWB and the WSN technologies. Proceedings of the 2014 International Conference on Indoor Positioning and Indoor Navigation (IPIN).

[24] Tan K.M., Law C.L. GPS and UWB Integration for indoor positioning. Proceedings of the 2007 6th International Conference on Information, Communications & Signal Processing.

[25] Kok M., Hol J.D., Schön T.B. (2015). Indoor Positioning Using Ultrawideband and Inertial Measurements. IEEE Trans. Veh. Technol..

[26] Cebrian Á., Bellés A., Martin C., Salas A., Fernández J., Arribas J., Vilà-Valls J., Navarro M. Low-Cost Hybrid GNSS/UWB/INS Integration for Seamless Indoor/Outdoor UAV Navigation. Proceedings of the 32nd International Technical Meeting of the Satellite Division of The Institute of Navigation (ION GNSS+ 2019).

[27] Navarro M., Arribas J., Vilà-Valls J., Casademont J., Calveras A., Catalán M. Hybrid GNSS/INS/UWB Positioning for Live Demonstration Assisted Driving. Proceedings of the 2019 IEEE Intelligent Transportation Systems Conference (ITSC).

[28] AGAVE (2008): Project Final Report. https://cordis.europa.eu/project/id/17668/it.

[29] Decuir J. (2004). Two Way Time Transfer Based Ranging. Contrib. IEEE.

[30] Groves P.D. (2008). GNSS, Inertial and Multisensor Integrated Navigation Systems.

[31] Dabove P., Manzino A.M. Kalman Filter as Tool for the Real-time Detection of Fast Displacements by the Use of Low-cost GPS Receivers. Proceedings of the 2nd International Conference on Geographical Information Systems Theory, Application and Management (GISTAM).

